# French Teeth

**Published:** 1857-03

**Authors:** 


### FRENCH TEETH.

We recently saw some teeth of French manufacture, at the well known Dental Depot of J. M. Brown, which, in regard to texture, excelled any teeth we have ever seen. They are plain incisors and canines, and of very perfect form. They possess most perfectly that life-like appearance and texture of living teeth. In that respect it would be impossible to distinguish them from the natural teeth if matched in color.

We do not know anything about their strength, or how they will *stand fire*, and some think that is an important item. In texture we should like to see our manufacturers imitate them. They can do it.

